# Yeast lysates carrying the nucleoprotein from measles virus vaccine as a novel subunit vaccine platform to deliver *Plasmodium* circumsporozoite antigen

**DOI:** 10.1186/s12936-017-1908-7

**Published:** 2017-06-29

**Authors:** Daria Jacob, Claude Ruffie, Chantal Combredet, Pauline Formaglio, Rogerio Amino, Robert Ménard, Frédéric Tangy, Monica Sala

**Affiliations:** 1 Institut Pasteur, Viral Genomics and Vaccination Unit, CNRS, UMR-3965, 28, Rue Du Dr Roux, 75015 Paris, France; 2 Institut Pasteur, Malaria Biology and Genetics Unit, 75015 Paris, France; 3 Institute of Molecular and Clinical Immunology, Otto-von-Guericke University, Magdeburg, Germany

**Keywords:** *Plasmodium berghei*, Malaria vaccine, CS antigen, *Pichia pastoris*, Yeast lysate, Measles virus, Nucleoprotein, Subunit vaccine

## Abstract

**Background:**

Yeast cells represent an established bioreactor to produce recombinant proteins for subunit vaccine development. In addition, delivery of vaccine antigens directly within heat-inactivated yeast cells is attractive due to the adjuvancy provided by the yeast cell. In this study, *Pichia pastoris* yeast lysates carrying the nucleoprotein (N) from the measles vaccine virus were evaluated as a novel subunit vaccine platform to deliver the circumsporozoite surface antigen (CS) of *Plasmodium*. When expressed in *Pichia pastoris* yeast, the N protein auto-assembles into highly multimeric ribonucleoparticles (RNPs). The CS antigen from *Plasmodium berghei* (PbCS) was expressed in *Pichia pastoris* yeast in fusion with N, generating recombinant PbCS-carrying RNPs in the cytoplasm of yeast cells.

**Results:**

When evaluated in mice after 3–5 weekly subcutaneous injections, yeast lysates containing N-PbCS RNPs elicited strong anti-PbCS humoral responses, which were PbCS-dose dependent and reached a plateau by the pre-challenge time point. Protective efficacy of yeast lysates was dose-dependent, although anti-PbCS antibody titers were not predictive of protection. Multimerization of PbCS on RNPs was essential for providing benefit against infection, as immunization with monomeric PbCS delivered in yeast lysates was not protective. Three weekly injections with N-PbCS yeast lysates in combination with alum adjuvant produced sterile protection in two out of six mice, and significantly reduced parasitaemia in the other individuals from the same group. This parasitaemia decrease was of the same extent as in mice immunized with non-adjuvanted N-PbCS yeast lysates, providing evidence that the yeast lysate formulation did not require accessory adjuvants for eliciting efficient parasitaemia reduction.

**Conclusions:**

This study demonstrates that yeast lysates are an attractive auto-adjuvant and efficient platform for delivering multimeric PbCS on measles N-based RNPs. By combining yeast lysates that carry RNPs with a large panel of *Plasmodium* antigens, this technology could be applied to developing a multivalent vaccine against malaria.

**Electronic supplementary material:**

The online version of this article (doi:10.1186/s12936-017-1908-7) contains supplementary material, which is available to authorized users.

## Background

Despite great progress in vaccine development [1], many heavy-burden diseases are still missing a vaccine, including malaria, AIDS and neglected tropical diseases that affect the poorest regions of the world resulting in reduced public health and serious socio-economic consequences. Therefore, developing safe, efficient and low-cost new vaccine platforms is of paramount importance. In this regard, the use of whole heat-inactivated yeast, a very cheap system to deliver recombinantly expressed antigens, is being explored [2–6]. For instance, several therapeutic vaccine candidates based on heat-inactivated *Saccharomyces cerevisiae* yeast expressing cancer or viral antigens are authorized by the FDA and show promising results in Phase I and II clinical trials [7].

In a previous study, a novel subunit vaccine platform based on antigen delivery within heat-inactivated *Pichia pastoris* yeasts was developed [6]. In addition to yeast-based delivery, the antigen was multimerized by its fusion to the nucleoprotein (N) of measles virus vaccine strain (MV) [8] expressed in the yeast cytoplasm. Multiple studies demonstrated the importance of presenting antigens to the immune system in structurally organized and repeated forms, for instance, by antigen association to VLPs (virus-like particles) or synthetic nanoparticles [9–11]. The measles virus N protein has a remarkable capacity to auto-assemble around local cytoplasmic RNA molecules in multiple expression systems (mammalian [12], bacterial [13], insect [14] or yeast [15] cells) and to generate helical, highly stable and multimeric ribonucleoparticles (RNPs), which resemble the RNP of MV [16]. To take advantage of such intrinsic properties, N-based RNPs the authors used to multimerize antigens of choice and deliver such antigen-carrying RNPs within whole heat-inactivated yeast [6].

To provide proofs of concept of this new subunit vaccine platform, the circumsporozoite protein (a major surface antigen of *Plasmodium berghei*, PbCS) was expressed in *Pichia pastoris* yeast in fusion with MV N, generating recombinant PbCS-carrying RNPs in the cytoplasm of yeast cells. Subcutaneous immunizations with whole recombinant *Pichia pastoris* yeasts expressing such RNPs induced a strong anti-PbCS humoral response, and upon challenge, delayed the onset of red blood cell infection by *P. berghei* and prolonged mouse survival [6]. Notably, these results were achieved in the C57Bl/6 mouse model, which is highly susceptible to *P. berghei* infection (ANKA strain) and subsequent development of cerebral malaria [17, 18].

To explore whether whole yeasts are essential for adjuvanting antigen immunogenicity, or yeast cell components associated to recombinant RNP nanoparticles are sufficient to provide adjuvancy, an alternative yeast vaccine formulation based on clarified lysates from yeasts expressing N-PbCS RNPs was evaluated in this study. These yeast lysates were clarified by removal of cellular membrane debris and nucleus, and they retained the RNPs and the cytoplasmic components of the yeast cells. Mice immunized with N-PbCS lysates showed parasitaemia reduction when lethally challenged with *P. berghei*, but not mice immunized with yeast expressing either N alone (naked RNPs) or PbCS alone (non-multimerized antigen). Sterile protection was achieved in groups immunized with high doses of adjuvanted and non-adjuvanted N-PbCS yeast lysates, demonstrating the efficacy of this vaccine formulation in the absence of external adjuvants.

## Methods

### Recombinant *Pichia* *pastoris* yeast used in the study

Whole recombinant *Pichia pastoris* yeast clones were obtained as previously described by stable and multiple integration of heterologous codon-optimized genes into the yeast genome, and, following methanol-induction, selection of the best expressing clone for the protein of interest [6]. Three recombinant yeast clones were generated expressing: (i) the measles virus (MV) nucleoprotein (N) alone (amino acid sequence corresponding to Schwarz vaccine strain [8]); (ii) the MV N protein fused in C-terminal with PbCS—the circumsporozoite (CS) antigen from *Plasmodium berghei*, ANKA strain; and (iii) the PbCS antigen alone. The level of expression of recombinant proteins in the selected clones was analysed by quantitative western blot and resulted in 870 ng/YU for N, 12 ng/YU for N-PbCS and 16 ng/YU for PbCS [6]. Expression of N in *Pichia pastoris*, as well as in other eukaryotic or prokaryotic expression systems, leads to formation of ribonucleoprotein nanoparticles (RNPs) localized in the cytoplasm of the yeast cell [12, 13, 15]. Fusion of the PbCS antigen to N was shown to retain RNP formation and resulted in recombinant RNPs presenting PbCS antigen on their surface (N-PbCS RNPs) [6].

### Preparation of clarified yeast lysates

Yeast cultures were induced for 54 h in the BMM medium (Invitrogen), supplemented every 24 h with 0.5% methanol. At 54 h, cultures were arrested and 250 YU (YU—yeast unit—10^7^ yeast cells) were pelleted in 2 ml Eppendorf tubes and washed twice in PBS to remove residual methanol. Yeast pellets were then resuspended in 100 μl of Breaking Buffer (50 mM sodium phosphate pH7.4, 1 mM EDTA, 5% glycerol and EDTA-free protease inhibitor cocktail (c*O*mplete, 11 873 580 001, Roche), 100 μl of acid-washed glass beads (425–600 μm; G8772, Sigma-Aldrich) were added, and samples were vortexed for 4 min at 4 °C. Yeast debris and glass beads were removed by centrifugation at 134*g* for 10 min at 4 °C, supernatants were transferred into new tubes, centrifuged at 371*g* for 15 min at 4 °C, and retransferred into new collection tubes. Obtained clarified lysates were pooled and/or supplemented with PBS to obtain the desired equivalent of yeast (30–300 YU) in 100 μl for mice immunization. Aluminum hydroxide gel (A8222, Sigma-Aldrich) was added at 1 μg/μl concentration.

### Western blot analysis of antigen concentration

250 YU aliquots from N or N-PbCS cultures were lysed and clarified as described above and stored at −80 °C for western blot (WB) analysis of antigen concentration after each immunization. Stored samples contained 28 μl of N-PbCS lysate or of a 1/125 dilution of N lysate, 10 μl of XT Sample Buffer 4× (161-0791, Bio-Rad) and 2 μl of XT Reducing Agent 20× (161-0792, Bio-Rad). WB was performed as previously described [6] in denaturing conditions on 4–12% Bis–Tris polyacrylamide gels with XT MOPS buffer (Criterion 345-0123, Bio-Rad) using the Color Plus Prestained Protein Ladder (7-200 kDa; P7711 BioLabs). Anti-N monoclonal antibody (3E1, Abnova) was used at 1/2000 dilution to detect N-PbCS and N proteins in yeast lysate samples.

### Mice immunization and antibody detection by ELISA

Six weeks old C56Bl/6 females were housed and manipulated according to the European Directive N° 2010/63/UE. The experimental protocol was submitted and approved by the Ethic Comity Ile-de-France – Paris 1 (N° 2012-0009). All the experimenters had a regulatory authorization for animal handling delivered by the accredited French authorities and accepted by the Animal Facility of Institut Pasteur, Paris. All efforts were made to minimize animal suffering and to reduce the number of animals used. Mice were injected subcutaneously with 100 μl of freshly prepared vaccine formulations in correspondence of inguinal lymph nodes. Mouse bleedings were collected before each immunization, sera were separated from blood samples and analysed for anti-PbCS antibodies by ELISA as previously described [6]. Titers were determined as the inverse of the highest sample dilution for which the OD_450nm/620nm_ signal was greater than the cut off (the mean optical density plus 3 times the standard deviation of pre-immune control sera from mice under study).

### Mice challenge by *Plasmodium berghei*

Lethal doses of freshly dissected *P. berghei* ANKA GFP^+^ sporozoites (8 × 10^3^–10^4^) were used to challenge mice intradermally at day 43 (2 weeks after the last immunization) as previously described [6]. After challenge, mice were monitored daily during 2 weeks for clinical signs of neurological disorder, then twice a day up to day 29 for the animals that recovered from initial symptoms. To determine the clinical benefits of vaccination and avoid bias, mice were not euthanized before day 15. Beyond day 15, surviving mice displayed constant hyper-parasitaemia, but did not show any predictive symptoms until sudden death. Identified moribund mice were euthanized by CO_2_ treatment in an appropriate chamber. Blood samples (2 μl) were collected from day 3–7, diluted in 600 μl of PBS and percentage of GFP^+^ infected red blood cells (iRBC) was determined by Fluorescence Activated Cell Sorting (FACS; MacsQuant, Miltenyi Biotec). Mann–Whitney nonparametric test was performed using GraphPad Prism version 5.0b for Mac OS X, GraphPad Software, San Diego California USA.

## Results

### Yeast lysates of *Pichia pastoris* expressing N-PbCS RNPs are immunogenic

In our previous work, we showed that whole recombinant *Pichia pastoris* yeast expressing N-PbCS RNPs elicited significant reduction of parasitaemia and decreased clinical damages associated with a highly stringent *P.* *berghei* infection in C57Bl/6 mice [6]. This provided a proof of concept of the intrinsic adjuvancy of RNPs expressed in whole heat-inactivated yeast cells for antigen delivery. It was then necessary to evaluate whether integrity of the yeast cell is indispensable to elicit yeast-based adjuvancy and protection in the same model. To address this question, whole recombinant yeasts lysates expressing N-PbCS, N, or PbCS were prepared by mechanical lysis and partial clarification. Partial clarification of lysates allowed eliminating yeast nuclei and major membrane layers while retaining most yeast cell components including recombinant antigen-carrying RNPs. In a previous study, the presence of highly multimeric RNPs in yeast lysates was demonstrated by ultracentrifugation and electron microscopy [6]. These lysates were evaluated in mice for their immunogenicity. All experimental parameters for yeast inoculation or vaccine dose formulation (i.e. time, temperature, volume of induction and lysis of yeast cultures) were standardized in order to minimize variability among experiments. Western blot analysis of clarified lysates prepared according to the standardized protocol from five independent yeast cultures proved that the established protocol was highly reproducible (Additional file 1: Figure S1).

Prior to each immunization, yeast lysates were prepared from freshly cultured yeast and aliquoted to obtain the equivalent of 30 YU (1 YU = 10^7^ cells) in 100 μl injection-volume. Five immunization groups were included in the first study (Experiment I): mice immunized with lysates of WT yeasts or recombinant yeasts expressing N-PbCS, N, or PbCS antigens with a naive control group of unvaccinated mice. N and PbCS control groups were immunized with the same amount of lysate and of respective antigen as indicated for the N-PbCS group (Table 1, Experiment I). Here and in the following experiments, the molar quantity of N or PbCS antigen in yeast (pmol/YU) was adjusted to the level of N-PbCS by “dilution” of recombinant yeast with WT yeast. The N and WT control groups consisted of six C56Bl/6 mice, while the N-PbCS, PbCS, and naive groups were comprised of eight mice.

**Table 1:** Groups of mice, vaccine formulations, doses and administration protocols used in Experiments I, II and III

Group	Yeast lysate dose (YU)	Adjuvant	Number of mice	Number of immunizations
Experiment I				
N-PbCS	30	No	8	5
PbCS	30^a^	No	8	5
N	30^a^	No	6	5
WT	30	No	6	5
Naive	/	/	8	/
Experiment II				
N-PbCS	30	No	6	3
N-PbCS	30	No	6	5
N-PbCS	150	No	6	5
N-PbCS	300	No	6	5
N	30^a^	No	6	3
N	30^a^	No	6	5
N	150^a^	No	6	5
N	300^a^	No	6	5
WT	30	No	4	3
WT	30	No	4	5
WT	150	No	4	5
WT	300	No	4	5
Naive	/	/	8	/
Experiment III				
N-PbCS	50	Yes	6	3
N-PbCS	100	Yes	6	3
N-PbCS	150	Yes	6	3
N	50^a^	Yes	6	3
N	100^a^	Yes	6	3
N	150^a^	Yes	6	3
WT	150	Yes	6	3
Alum	/	Yes	6	3
Naive	/	/	6	/

^a^Diluted with WT yeast according to the following calculation:					
	Expression level, ng/YU	Mol. weight, g/mol	Expression level, pmol/YU	Folds diluted with WT to match N-PbCS	Antigen dose in 30 YU, ng
N-PbCS	12	91,32	131	/	360
PbCS	16	33,24	481	3,7	130
N	870	58,08	14,979	114,0	230

1 YU = 10^7^ cells

**Fig. 1:**
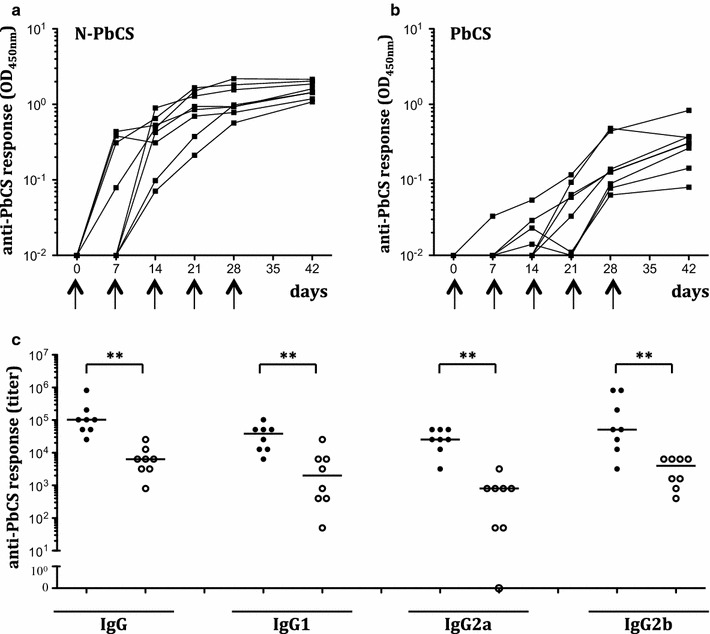
Humoral anti-PbCS responses in immunized mice. Kinetics of anti-PbCS IgG responses elicited in mice after immunization with 30YU of N-PbCS (**a**) or PbCS (**b**) non-adjuvanted yeast lysate. OD_450 nm_ are expressed in log_10_ scale.* Black arrows* indicate injection schedule. Mouse sera were analysed at 1/10^3^ dilution. **c** Isotyping of humoral anti-PbCS IgG responses at day 42 in mice immunized with 30 YU of N-PbCS (*black circles*) or PbCS (*white circles*) yeast lysates.* Bars* correspond to median values per group. *Asterisks* indicate significant median differences (*two symbols* for p < 0.005, Mann–Whitney nonparametric test)

All mice were subcutaneously injected at days 0, 7, 14, 21, and 28 (5 weekly immunizations). Before each immunization, blood samples were taken to follow the anti-N and anti-PbCS antibody responses. In the N-PbCS group, two injections at a 1-week interval were sufficient to elicit a detectable anti-PbCS antibody response in all mice (Fig. 1a), while in the PbCS group four injections were necessary (Fig. 1b). Following the fifth injection at day 28, both groups of mice attained plateauing levels of anti-PbCS responses; however, in the N-PbCS group the response was much higher than in PbCS mice. At day 42 (the pre-challenge time point), there was a significant difference (p < 0.005) in anti-PbCS IgG titers in N-PbCS and PbCS groups: 10^5^ and 7 × 10^3^ median values, respectively (Fig. 1c). No anti-PbCS antibodies were detected in mice immunized with N or WT yeast and in naive mice.

In the *P. berghei*—C57Bl/6 mice infection model, direct evidence of anti-PbCS T cell response implication could not be provided, as no T cell epitopes from PbCS have been identified [19]. Therefore, elicitation of T cell responses was evaluated indirectly by anti-PbCS IgG isotyping [20]. In both groups, the IgG isotype profile indicated elicitation of the Th1 (IgG2a and IgG2b) and Th2 (IgG1) arms of the cellular immune response; however, the N-PbCS group titers reached one to two-log higher isotype titers (Fig. 1c).

Altogether, these observations show that N-based RNPs delivering PbCS on their surface induce anti-PbCS specific antibody responses at earlier time points and at significantly higher titers than the same amount of monomeric PbCS antigen (128 ng/dose), while PbCS multimerization does not alter isotype profiles of anti-PbCS humoral responses.

**Fig. 2:**
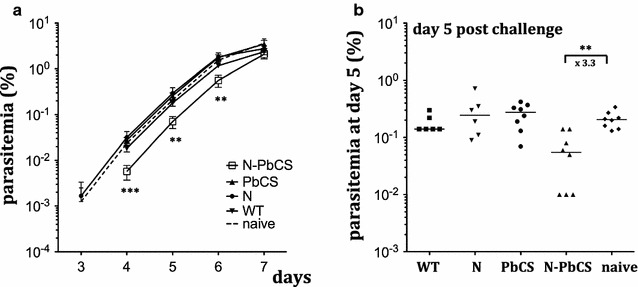
Experimental challenge of immunized mice (Experiment I). **a** Mean and standard deviation log_10_ values of parasitaemia in mice immunized with 30 YU of N-PbCS, PbCS, N, or WT non-adjuvanted yeast lysates, and in non-immunized mice following infection with 8 × 10^3^ GFP^+^ *P. berghei* sporozoites at day 0. *Asterisks* indicate significant median differences (two symbols for p < 0.005, three for p < 0.0005; Mann–Whitney nonparametric test). **b** Parasitaemia at day 5 post challenge. *Bars* correspond to medians. *Asterisks* indicate significant median differences (*two symbols* for p < 0.005, Mann–Whitney nonparametric test). Decrease in parasitaemia median value in N-PbCS immunized group in comparison with the naive group is indicated in folds

At day 42, mice were intradermally challenged with 8 × 10^3^ GFP^+^ *P. berghei* sporozoites and tail snip blood samples were taken daily from day 3 to 7 to analyse the effect of immunization on infection. Parasitaemia (percentage of infected red blood cells (iRBCs) to total circulating red blood cells) was monitored by quantifying the GFP^+^ iRBCs in the blood. In this experimental model, around 48–52 h after infection, parasites that have developed in the liver are released in the blood and iRBCs can be detected by flow cytometry at earliest on day 3 and theoretically at the latest on day 7, unless sterile protection against sporozoites and hepatic stages is achieved. In addition, days 3–7 corresponds to the interval when the iRBCs/RBC ratio increase is linear and this ratio is, therefore, used to compare protection efficiency between different groups. By day 7, a first plateau is then reached, followed by sequential peaks induced by infection bursts. Following challenge, mice immunized with N-PbCS yeast lysates displayed a significant delay in parasitaemia, which was detected by the decrease in iRBC percentage with respect to controls groups immunized with PbCS, N, and WT, and non-immunized mice at days 4, 5, and 6 after challenge (Fig. 2a). Notably, at day 5 post infection, the N-PbCS group showed a reduction of 3.3 times in median parasitaemia with respect to the naive group, in which parasitaemia was comparable to that in PbCS, N, and WT groups (Fig. 2b). Therefore, in these experimental conditions, immunization with N-PbCS yeast lysates could not prevent infection; however, it induced a significant delay in parasitaemia by reducing hepatic parasite load. Vectorization of the PbCS antigen on multimeric N-based RNPs nanoparticles was essential for providing significant parasitaemia delay, as such an effect was not achieved in mice immunized with monomeric PbCS in yeast lysates.

Typical surviving patterns were observed across all groups with two mortality peaks at days 7–12 due to cerebral malaria and 23–27 due to anaemia. No benefit in overall survival was observed in mice immunized with N-PbCS comparing to PbCS, WT and naive groups (Additional file 2: Figure S2). Three out of six mice in the N-PbCS group escaped cerebral malaria and survived over 20 days, while in the N group five out of six mice did, however, relatively low numbers of mice per group do not allow concluding whether the difference in survival was statistically significant.

### Escalating doses of N-PbCS in yeast lysates induce higher antibody titers and improve protective efficacy

To test whether increasing the PbCS antigen quantity per injection would increase the immune response, we designed a second experiment where groups of six *C57BL/6* mice were injected with 30, 150, and 300 YU doses (Table 1, Experiment II). An additional N-PbCS 30 YU group of six mice was immunized at days 0, 14, and 28 in order to compare protocols with 5 weekly and three bi-weekly injections. 150 and 300 YU doses were produced after pooling several yeast lysates, while maintaining the final injection volume of 100 μl.

**Fig. 3:**
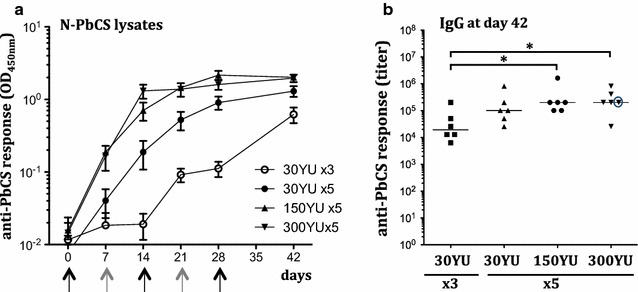
Humoral anti-PbCS responses in immunized mice. **a** Kinetics of anti-PbCS IgG responses elicited in mice immunized with 30, 150 and 300 YU of N-PbCS non-adjuvanted yeast lysates. *Curves* represent group mean value with SEM. OD_450nm_ are expressed in log_10_ scale. “x3”—three bi-weekly immunizations; “x5”—five weekly immunizations. Mouse sera were analysed at 1/10^3^ dilution. **b** Isotyping of humoral IgG responses at day 42 in mice immunized with 30, 150, and 300 YU of N-PbCS non-adjuvanted yeast lysates. *Bars* correspond to median values per group. *Asterisks* indicate significant median differences (*one symbol* for p < 0.05, Mann–Whitney nonparametric test). Antibody titer of the non-parasitized mouse is encircled

In groups immunized with 5 weekly doses of 150 and 300 YU of N-PbCS yeast lysates, a single injection was sufficient to trigger detectable levels of anti-PbCS antibodies, and two (for 300 YU) to three (for 150 YU) injections induced plateauing levels (Fig. 3a). With the lower 30 YU dose, two injections were required to induce anti-PbCS antibodies, which increased with subsequent injections. All groups with 5 weekly injections had comparable N-PbCS antibody responses at day 42, with IgG titers reaching the plateauing level of 3 × 10^5^ (Fig. 3b). Therefore, 5 weekly immunizations with 30 YU N-PbCS yeast lysate (128 ng/dose; 640 ng/protocol; Table 1, Experiment II) were sufficient to induce a plateauing level of anti-PbCS antibodies by day 42, and this response was not increased by 5- or 10-fold higher doses (i.e. 1.3 μg/dose; 6.4 μg/protocol in the 300 YU group). Moreover, comparison between three bi-weekly and five weekly immunizations with the same antigen/lysate dose of 30 YU showed that weekly injections were necessary to trigger higher and earlier (from day 7) anti-PbCS antibodies (Fig. 3a). Five weekly immunizations as well as higher antigen doses were also beneficial for inducing increased levels of IgG1, IgG2a, and IgG2b isotypes (Additional file 3: Figure S3).

**Fig. 4:**
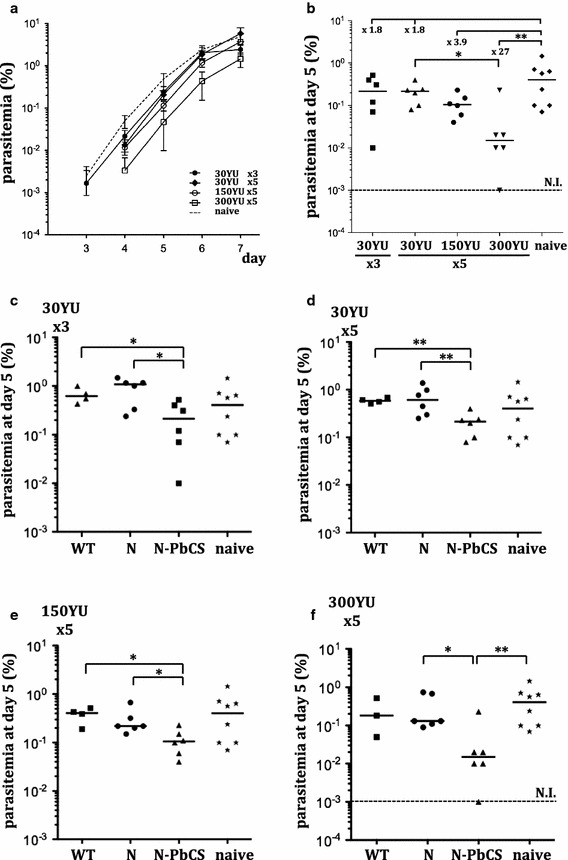
Immunogenicity of N-PbCS at 30, 150 and 300 YU in yeast lysates. **a** Mean and standard deviations log_10_ values of parasitaemia in mice immunized with N-PbCS at different doses and regimens and in non-immunized mice (naive) following infection with 10^4^ GFP^+^ *P. berghei* sporozoites. **b** Parasitaemia at day 5 post challenge. *Bars* correspond to medians. Decrease in parasitaemia median value in immunized groups in comparison with the naive group is indicated in* folds*. The non-parasitized mouse was excluded from calculation. **c**–**f** Groups of mice immunized with the following formulations and non-immunized mice from the same experiment: **c** 30 YU N-PbCS, N or WT yeast lysates 3 times bi-weekly; **d** 30 YU N-PbCS, N or WT yeast lysates 5 times weekly; **e** 150 YU N-PbCS, N or WT yeast lysates 5 times weekly; **f** 300 YU N-PbCS, N or WT yeast lysates 5 times weekly. *Bars* correspond to medians. *NI* not infected over 10 days post challenge (value below the limit of detection). *Asterisks* indicate significant median differences (*one symbol* for p < 0.05 and two for p < 0.005; Mann–Whitney nonparametric test). The non-parasitized mouse was excluded from calculation

Following challenge with 10^4^ GFP^+^ *P. berghei* sporozoites at day 42, blood samples were collected and analysed as describe above. Among the groups of mice immunized with N-PbCS yeast lysates, parasitaemia delay from days 3–7 was dose-dependent and proportional to the dose and amount of injections (Fig. 4a). While three bi-weekly and five weekly immunizations with 30 YU N-PbCS yeast lysate produced only a 1.8-fold decrease in median parasitaemia at day 5 post infection compared to the naive group of mice, 5 weekly immunizations with 150 and 300 YU resulted in a 3.9-fold and as much as 27-fold decrease in median parasitaemia at day 5, respectively (Fig. 4b). Notably, there was statistically significant difference between mice immunized five times with 30 and 300 YU at day 5 after challenge. All together, this data shows that higher doses of PbCS delivered on N-based RNPs had an increasingly beneficial effect against early blood stage *P.* *berghei* infection . A slight divergence in parasitaemia delay compared to Experiment I with 5 weekly immunizations of 30 YU (×3.3; Fig. 2b) was observed and was due to different availability of the sporozoite preparation (8 × 10^3^ sporozoites/mouse in Experiment I vs. 10^4^ in Experiment II) and divergence in lot infectivity. Noteworthy, higher doses of yeast lysates (150 and 300 YU) were prepared by pooling several yeast lysate preparations, as pure RNP concentration was not technically achievable. As a result, the “yeast background” was increased proportionally in these formulations. Nevertheless, control groups of mice immunized with equivalent doses of N and WT yeast lysates were present all along the study, and no significant effect of N and WT yeasts on infection prevention was detected (Fig. 4c–f). In mice immunised with 30YU N-PbCS weekly or bi-weekly, increased resistance to cerebral malaria was observed, as seen by a higher number of N-PbCS-immuzied mice surviving over 15 days comparing to N and WT groups; however, this effect was not present in groups with higher doses (Additional file 4: Figure S4).

Notably, one mouse in the 300 YU group maintained negative parasitaemia during all the study follow-up (30 days post-challenge) and developed no clinical signs of malaria, indicating sterile protection against *P. berghei* infection, while typical surviving profiles in all other mice across N-PbCS immunized groups were observed with two mortality peaks at days 7–12 and 23–27 (Additional file 6: Figure S6). This mouse was regularly monitored for infection by classical blood smear analysis and no iRBCs were identified in its blood over 4 months post-challenge. The protected mouse displayed levels of anti-PbCS antibodies comparable to those of the other non-protected mice from the same group (Fig. 3b; Additional file 3: Figure S3). At day 118, this mouse was re-challenged with 10^4^ sporozoites together with a control group of 3 naive mice of the same age. It’s N-PbCS antibody level was measured at day 113 and was found comparable to the level at day 42 before the first challenge. Protection acquired by the first immunization could not protect the mouse from the second challenge, as the parasitaemia kinetics in this mouse was identical to that of non-immunized control mice (Additional file 5: Figure S5).

### Immunizations with alum-adjuvanted N-PbCS yeast lysates provide sterile protection in a third of immunized mice

The sterile protection obtained in the 300 YU group was unpredictable by anti-PbCS humoral responses elicited in the protected mouse before challenge, and was a rare event in the study group. A preliminary experiment on three mice showed that immunization with 30 YU N-PbCS lysates adjuvanted with alum (aluminum hydroxide) induced anti-PbCS IgG antibodies comparable to those induced by the equivalent non-adjuvanted lysate formulation (Additional file 6: Figure S6). However, anti-PbCS IgG1, IgG2a, and IgG2b isotype profiles differed in these formulations. Increased titers of IgG1 and decreased titers of IgG2a induced by the alum-adjuvanted lysate formulation indicated strong potentiation of Th2 responses by alum (Additional file 6: Figure S6), in accordance with its intrinsic antibody stimulating properties [21]. Therefore, at the next step, the implication of anti-PbCS antibody enhancement by alum adjuvant on clinical benefit and protection was evaluated in the same stringent infection model.

Injection doses of 50, 100, and 150 YU of yeast lysates (215, 430, and 645 ng of PbCS per injected dose, respectively) were supplemented with 50 μg of alum per dose (Table 1, Experiment III). Groups of six mice were immunized with three escalating doses of either N-PbCS yeast lysate or, for the control groups, N, or WT lysates. Two other groups of six mice immunized with alum alone and naive mice were added as controls. Preliminary tests using alum-adjuvanted yeast lysates demonstrated that weekly administrations induce inguinal inflammations, evolving in most cases into skin ulcers, despite alternation of injection sites between both sides of the abdomen (these ulcers spontaneously recovered once the immunization aborted). For this reason, lysates supplemented with alum were injected only by three bi-weekly immunizations, and 2 weeks after the last immunization, mice were challenged with 10^4^ GFP^+^ *P.* *berghei* sporozoites.

**Fig. 5:**
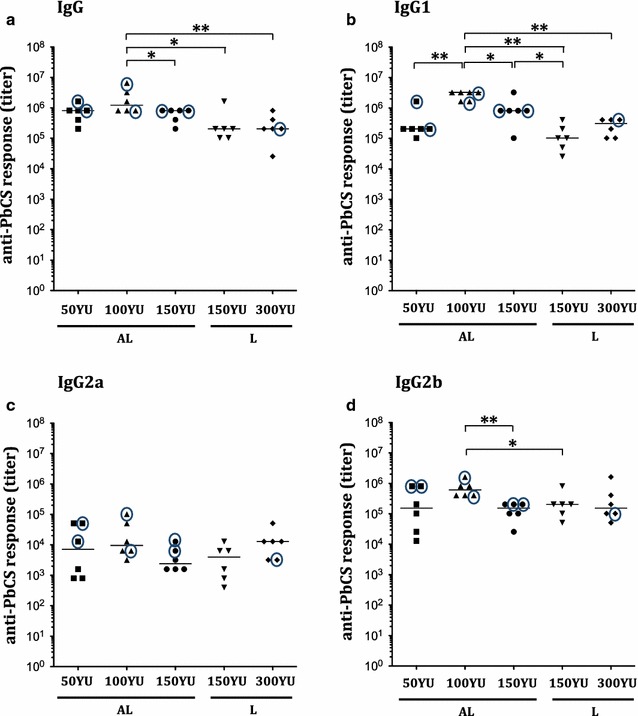
Immunogenicity of N-PbCS adjuvanted and non-adjuvanted yeast lysates. Isotyping of humoral IgG responses at day 42: IgG (**a**), IgG1 (**b**), IgG2a (**c**), and IgG2b (**d**). *Bars* correspond to median values per group. Antibody titers of the non-parasitized mice are encircled. *Asterisks* indicate significant median differences (*one symbol* for p < 0.05 and two for p < 0.005; Mann–Whitney nonparametric test). *L* non-adjuvanted yeast lysates, 5 weekly immunizations (Experiment II), *AL* alum-adjuvanted yeast lysates, three bi-weekly immunizations (Experiment III)

**Fig. 6:**
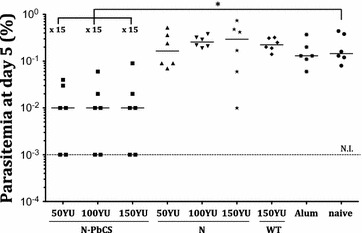
Comparison in parasitaemia at day 5 post-challenge. *AL* alum-adjuvanted yeast lysates, three bi-weekly immunizations (Experiment III), *L* non-adjuvanted yeast lysates, 5 weekly immunizations (Experiment II). *Alum* group of mice immunized with alum only. *Bars* correspond to medians among parasitized mice in the group. *NI* not infected over 10 days post challenge (value below the limit of detection). *Asterisk* indicates significant median differences (*one symbol* for p < 0.05 and two for p < 0.005, Mann–Whitney nonparametric test). Decrease in parasitaemia median value in immunized groups in comparison with the respective naive group from the same experiment is indicated in* folds*

All three doses of alum-adjuvanted N-PbCS yeast lysates induced the same kinetics of anti-PbCS antibody response, which reached the plateau after the second immunization by day 21, and remained at this level until the pre-challenge time point (Additional file 7: Figure S7). After infection, one-third of all mice immunized by N-PbCS alum-adjuvanted formulations were sterilely protected (as demonstrated by their healthy condition and survival over day 30 post infection) (Additional file 8: Figure S8), while the other four mice had a significant decrease in parasitaemia comparing to control groups (Fig. 6). Indeed, median parasitaemia across groups immunized with 50, 100, and 150 YU of alum-adjuvanted N-PbCS yeast lysates was 15-fold lower than in naive control mice at day 5 after challenge. While complete protection of all mice in N-PbCS groups could not be achieved, the minimal dose (50 YU) was sufficient to induce PbCS-mediated protection in several individual mice. As previously, levels of anti-PbCS IgGs or their isotypes were not predictive of sterile protection (Fig. 5).

## Discussion

Yeast cells represent an established bioreactor for producing recombinant proteins for pharmaceutical use [22, 23]. Notably, for vaccination purposes, delivery of antigens directly within heat-inactivated yeast cells has recently become attractive due to adjuvancy provided by the yeast cell [3, 7]. In this study, yeast lysates were evaluated as a platform for delivery of antigens multimerized by means of the measles virus nucleoprotein (N). The N protein, to which the antigen is fused, is expressed in the cytoplasm of *Pichia pastoris* yeast, where it spontaneously forms multimeric antigen-covered RNPs [6]. As purification of intracellularly produced RNPs would represent a technically complex and expensive process (which, moreover, could damage the integrity of these multimeric particles), RNP delivery within yeast lysates was performed. Yeast lysate preparation is a common and highly reproducible technique, which allows obtaining clarified yeast extracts that contain yeast cytoplasmic components. By immunizing mice with RNPs within clarified yeast lysates, the adjuvancy properties that internal yeast components add to the RNP-associated antigen were shown, and protective efficacy of this vaccine formulation in vivo in a malaria infection model was demonstrated.

The study was performed using the CS antigen from *P. berghei* (PbCS) in a stringent rodent malaria model (C57Bl/6 mice) challenged with a lethal dose of *P. berghei* sporozoites [17]. As the PbCS antigen possesses moderate intrinsic immunogenicity and is not fully protective in a monomeric vaccine formulation [24, 25], it allowed us to evaluate doses effects and the intrinsic potency of the yeast lysate formulation for RNP delivery.

Contrarily to whole yeasts, yeast lysates do not contain cell walls and, therefore, no PAMPs (pathogen-associated molecular patterns) that are located on the surface of the yeast cell and serve as danger signals for recognition by cells of the immune system [26]. Yet, yeast lysates represent a formulation of yeast proteins and nucleic acids of foreign nature, which may as well be recognized as danger signals by mammalian cells. Our results demonstrate that delivery of a relatively low amount of monomeric PbCS antigen adjuvanted by yeast lysate (360 ng of PbCS) induced detectable levels of anti-PbCS humoral response after two to four injections, although to a lower extent than multimeric N-PbCS at the same antigen dose. Interestingly, in our previous studies, the same amount of monomeric PbCS delivered in whole heat-inactivated yeast in the same immunization protocol did not induce any detectable levels of anti-PbCS antibodies during 42 days of immunization follow-up [6]. Moreover, immunization with 30 YU of N-PbCS yeast lysates (Fig. 1c, median value 10^5^ at day 42) induced about tenfold higher titers than 30 YU of N-PbCS delivered in heat-inactivated yeast (median value 2 × 10^4^ at day 42, [6]). These data indicate that adjuvancy properties of whole heat-inactivated yeast and yeast lysates are different, and that yeast lysates are advantageous as an adjuvant for inducing strong humoral responses against the delivered antigen.

With regards to the previous study [6], the non-specific protective effect of N delivered in whole yeast was not reproduced in yeast lysates (even at higher doses of the vaccine). It was observed that immunization with heat-inactivated yeast created a visible inflammation at the injection site (located in the proximity of the inguinal lymph node), while immunization with any dose of adjuvanted or non-adjuvanted yeast lysates gave no visible inflammation. It is possible that the inflammation induced by N-expressing heat-inactivated yeast resulted in non-specific activation of innate immune responses, especially at the level of the liver, providing a non-specific helper effect to the anti-Pb specific immune response (possibly by creating a cytokine environment favourable for adaptive responses against infection). In case of yeast lysates, no such effect was observed.

In accordance with other studies that demonstrate the advantage of multimerized antigen delivery [27, 28], RNPs proved to be a potent platform for increasing PbCS immunogenicity. Yeast lysates carrying non-multimerized PbCS induced significantly lower PbCS antibody responses, comparing to N-PbCS yeast lysates, in which the antigen was multimerized on RNPs (Fig. 1). While same amounts of multimerized and non-multimerized PbCS in yeast lysates were used in Experiment I, the latter were not protective against *P.* *berghei* challenge (Fig. 2). On the contrary, multimerized PbCS induced strong humoral responses and provided parasitaemia delay, demonstrating the immunogenic relevance of antigen multimerization by fusion to RNPs. While this formulation was not sterilely protective against *P.* *berghei* infection, it significantly delayed parasitaemia development, proving that vaccine-induced parasite arrest has occurred during the pre-erythrocytic phase of the infection. This effect was obtained by 5 weekly immunizations with lysates that contain 360 ng of PbCS per dose (a total of 1800 ng per protocol), which is a relatively low amount of antigen, in comparison with several micrograms of the CS antigen combined with an adjuvant used in mice studies of the RTS,S malaria vaccine [29, 30]. These observations confirm the data of our previous studies showing that monomeric PbCS delivered within heat-inactivated whole yeast did not provide any protection against challenge, while N-PbCS in the same formulation induced parasitaemia reduction [6]. Therefore, the benefit of antigen multimerization on RNPs was attested for both yeast lysate and heat-inactivated yeast formulations.

The dose of antigens delivered within heat-inactivated yeast is limited by the antigen’s expression level, as injections with such formulations can be tolerated up to a certain threshold. Preliminary tests have showed that 30 YU is the maximal amount of yeast that can be administered to mice without causing severe inflammation at the injection site. Yeast lysates, however, offer the possibility of injecting higher doses by pooling several yeast lysates and obtaining a maximum equivalent of 300 YU per dose in a volume of 100 µl, which was in accordance with regulations for weekly immunizations in mice and was well tolerated in our experiments. Evaluation of several vaccine doses allowed us to establish the dose-dependent efficiency of the anti-PbCS immune response, as increasing doses of non-adjuvanted N-PbCS yeast lysates (30, 150, and 300 YU) induced increasing parasitaemia delay. Moreover, one animal out of six in the group immunized with the highest dose (300 YU) was parasite-free during all the follow-up and developed no clinical symptoms of infection, indicating sterile protection. As the titer of anti-PbCS antibody response in this mouse at the pre-challenge point is equal to the group’s median value, cellular immune responses must have been implied in inducing sterile protection. Nevertheless, this experiment demonstrated that protection provided by anti-PbCS immune responses induced by repetitive immunizations with N-PbCS yeast lysates is a function of the PbCS antigen dose.

Comparable plateauing titers of anti-PbCS IgG titers were observed among all mice immunized with 50, 100, or 150 YU of N-PbCS lysates adjuvanted with alum (a total of 1800, 3600, and 5400 of PbCS per protocol) (Fig. 5a). Only two out of six mice (33%) in each of these groups showed sterile protection (please rephrase), demonstrating the limited role of elicited anti-PbCS antibodies on protection against *Plasmodium* infection. This result is consistent with multiple studies that evaluated monovalent CS-based malaria vaccines [24]. Notably, among the four remaining non-sterilely protected mice in these groups, comparable rates in parasitaemia decrease were observed (Fig. 6). This suggests that the level of protection reached (33%) was the greatest possible to be achieved in the given malaria infection model with a monovalent CS-based subunit vaccine. Notably, the same level of parasitaemia decrease was observed in mice immunized with the highest dose of non-adjuvanted N-PbCS lysate from Experiment II (300 YU, a total of 18,000 ng of PbCS per protocol). Hence, by adding alum adjuvant, lower and less frequent doses of N-PbCS were sufficient to provide an equivalent protection to that achieved by a higher dose of non-adjuvanted lysate. As the results of Experiment II suggest, protective efficacy provided by non-adjuvanted N-PbCS yeast lysates was proportional to the dose of PbCS antigen within the formulation. Therefore, it was demonstrated that the highest dose of non-adjuvanted N-PbCS yeast lysate induced the maximum level of protection that could be obtained in given experimental settings, eliminating the necessity of using alum adjuvant in the formulation.

This study provides proof of concept of recombinant RNPs delivered in yeast lysates as an efficient antigen delivery platform for preventive vaccines. Despite intrinsic moderate immunogenicity of the PbCS antigen, previously demonstrated in multiple antigen vectorization systems [24, 25], this novel vaccine platform succeeded in inducing significant benefit against a stringent malaria challenge by slowing down infection, alleviating clinical symptoms of the disease and providing sterile protection is subgroups of mice. However, considering the development of a malaria vaccine, higher rates of sterile protection have to be achieved in pre-clinical studies. The highly stringent infection model involving C57Bl/6 mice infected with *P. berghei* [17] was chosen to evaluate the efficiency of the vaccine platform in the most relevant conditions. So far, no study has shown 100% sterile protection in C57Bl/6 mice infected with *P. berghei* by means of a monovalent PbCS subunit vaccine. Complete sterile protection in C57Bl/6 mice can be induced by injection of whole irradiated, genetically-modified or chemically attenuated sporozoites, or by infection with intact sporozoites followed by anti-malaria drug treatment [31–34]. All these techniques rely on presenting the whole panel of *Plasmodium* antigens to induce fully protective immune responses. In the present study, 33% of mice were sterilely protected from *P. berghei* challenge upon immunization with 3 doses of alum-adjuvanted yeast lysates carrying N-PbCS RNPs at doses of 50, 100, or 150 YU, which correlates with the 33% protective efficacy of the RTS,S CS-based vaccine candidate in humans [25, 30]. This result is in accordance with observations that immune responses against the CS protein block parasite development in the liver, but are not sufficient to induce complete sterile protection, and, therefore, multivalency is a prerequisite for designing an efficient subunit malaria vaccine [35–37].

The carrier RNPs in the present antigen vectorization platform are constituted of the measles virus nucleoprotein N, which is a major antigenic component of the current live attenuated measles vaccine and induces high levels of antibodies, which are not protective against measles [38]. The measles vaccine is given to infants at 6–9 months after birth depending on endemicity levels of measles, as earlier vaccination is ineffective due to the presence of maternal antibodies [39]. As malaria represents most risk to children under 5 years of age, an anti-malaria vaccine should be administered to infants as early as possible. The possibility that pre-existing immunity (via maternal antibodies or vaccination) to N would decrease the efficiency of the RNP-based vaccine uptake cannot be ruled out, in case of RNP-based vaccine administration following anti-measles vaccination, and the reverse situation can also be assumed. A study on the nucleoprotein N of the RSV virus assembled into rings and carrying three copies of M2e (matrix protein-2 extra-domain) epitopes of the influenza virus, showed that induction of anti-M2e antibodies in mice was not impaired by pre-existing immunity to N [40]. Moreover, with the measles vaccine administered in two doses, immunity to measles after the prime dose does not impair the efficiency of a booster dose at 16–18 months, which is on the contrary known to enhance the elicited immune responses. Similarly, pre-existing immunity to measles in mice did not interfere with induction of antibody responses upon immunization with the recombinant measles virus vector carrying chikungunya virus antigens [41]. Despite these encouraging data indicating the lack of vaccine interference in the presence of measles pre-immunity, eventual impact of pre-existing immunity to N induced by the RNP-based vaccine or by measles virus vaccination should be addressed. This can only be evaluated experimentally in a mouse animal model susceptible to MV infection (hCD46 ± IFNα/βR−/− or CD460IFNAR) [8]. In case interference takes place indeed, it is possible to constitute RNPs from alternative nucleoproteins derived from distantly related Morbilliviruses.

The designed vaccine platform has the capacity of composing custom multivalent formulations. Identified promising antigens can be fused to N and expressed in yeast, which then can be lysed and mixed at desirable ratios to constitute a multivalent subunit vaccine. Identification of highly immunogenic *Plasmodium* antigens is a prerequisite for developing an efficient multivalent subunit vaccine based on the proposed biotechnological platform. The efficiency of this vaccine platform relies in antigen multimerization and auto-adjuvancy. Its relatively low cost of production (yeast culture preparation in simple medium and scalable yeast lysis technology) makes it attractive for developing a multivalent malaria vaccine and vaccines against other infectious diseases in developing countries.

## Conclusions

This study provides proof of concept of *Pichia pastoris* yeast lysates carrying the measles vaccine virus nucleoprotein as an efficient platform for multimerization and delivery of the *Plasmodium* CS protein. *Pichia pastoris* yeast lysates carrying ribonucleoparticles formed by N-PbCS induce strong anti-PbCS responses and delay parasitaemia onset in a dose-dependent manner. In a highly severe rodent malaria model, sterile protection in 33% of mice is achieved via three bi-weekly immunizations with yeast lysates carrying high doses of antigen (230–640 ng N-PbCS/dose) supplemented with alum adjuvant. Unprotected mice in these groups show significant parasitaemia reduction, which is comparable to that in mice immunized five time weekly with non-adjuvanted yeast lysates carrying 1280 ng N-PbCS/dose, demonstrating, therefore, the auto-adjuvant property of the developed platform. This study lays ground to developing multivalent malaria vaccines using combinations of highly immunogenic antigens assembled at desired concentrations and delivered via the proposed subunit vaccine platform.

## Additional files

**Additional file 1: Figure S1.** Western blot analysis of clarified yeast lysates prepared from *Pichia pastoris expressing* N-PbCS (A) or N (B) at five different time points. Yeasts were cultured in independently prepared batches of media and lysed according to the standardized protocol. Samples of yeast lysates (non-diluted for N-PbCS and diluted 1/125 for N lysates) were prepared for western blot analysis and stored at −80 °C. Anti-N monoclonal antibody was used to detect N-PbCS and N proteins. Despite the presence of protease inhibitors in the lysis buffer, reproducible patterns of proteolysis were observed in the N-PbCS fusion protein, as previously observed [6].

**Additional file 2: Figure S2.** Survival curves of mice immunized with 30 YU of N-PbCS, PbCS, N or WT yeast lysates in Experiment I after challenge with GFP + Pb sporozoites.

**Additional file 3: Figure S3.** Isotyping of humoral IgG responses at day 42: IgG1 (A), IgG2a (B), and IgG2b (C) in mice immunized with 30, 150 and 300 YU of N-PbCS non-adjuvanted yeast lysates. Bars correspond to median values per group. “x3”-three bi-weekly immunizations; “x5”-five weekly immunizations. Mouse sera were analysed at 1/10^3^ dilution. Asterisks (*) indicate significant median differences (one symbol for p < 0.05, two for p < 0.005, Mann–Whitney nonparametric test). Antibody titers of the non-parasitized mouse are encircled.

**Additional file 4: Figure S4.** Survival curves of immunized mice in Experiment II after challenge with GFP + Pb sporozoites. (A)-30 YU N-PbCS, N or WT yeast lysates 3 times bi-weekly; (B)-30 YU N-PbCS, N or WT yeast lysates 5 times weekly; (C)-150 YU N-PbCS, N or WT yeast lysates 5 times weekly; (D)-300 YU N-PbCS, N or WT yeast lysates 5 times weekly.

**Additional file 5: Figure S5.** Second challenge of the mouse that survived the first challenge in Experiment II. Log_10_ values of parasitemia in the mouse immunized with 300 YU N-PbCS yeast lysate that survived first infection at day 42 (“N-PbCS survivor”) and three naive mice at day 5 following infection at day 118 with 10^4^ GFP^+^ *P. berghei* sporozoites.

**Additional file 6: Figure S6.** Isotyping of humoral IgG responses at day 42 in mice immunized with 30 YU N-PbCS yeast lysates. L-non-adjuvanted lysate formulation administered 5 times weekly, AL-alum-adjuvanted lysate formulation administered 3 times bi-weekly. Bars correspond to median values per group.

**Additional file 7: Figure S7.** Kinetics of humoral anti-PbCS responses in immunized mice with N-PbCS yeast lysates adjuvanted with alum. OD_450nm_ are expressed in log_10_ scale. Arrows indicate immunization schedule. Mouse sera were analysed at 1/10^3^ dilution.

**Additional file 8: Figure S8.** Survival curves of immunized mice in Experiment III after challenge with GFP + Pb sporozoites. Mice were immunized 3 times bi-weekly with (A)-50 YU N-PbCS or N yeast lysates; (B)-100 YU N-PbCS or N yeast lysates; (C)-150 YU N-PbCS, N or WT yeast lysates: in comparison to mice immunized with the adjuvant alone (Alum group) and naive mice.
