# Supplementary figures and images for: Yeast lysates carrying the nucleoprotein from measles virus vaccine as a novel subunit vaccine platform to deliver Plasmodium circumsporozoite antigen
Source: Malar J. 2017 Jun 29;16:259. doi: 10.1186/s12936-017-1908-7 (PMC5492716; doi:10.1186/s12936-017-1908-7)

**A**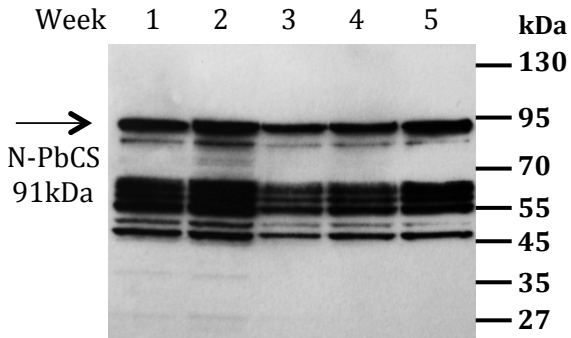**B**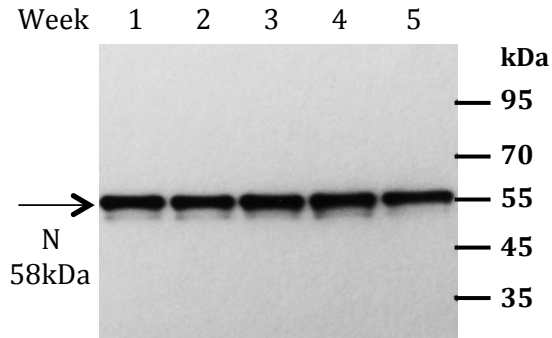

Supplement: Supplementary file 1 — Additional file 1: Figure S1. Western blot analysis of clarified yeast lysates prepared from Pichia pastoris expressing N-PbCS (A) or N (B) at five different time points. Yeasts were cultured in independently prepared batches of media and lysed according to the standardized protocol. Samples of yeast lysates (non-diluted for N-PbCS and diluted 1/125 for N lysates) were prepared for western blot analysis and stored at −80 °C. Anti-N monoclonal antibody was used to detect N-PbCS and N proteins. Despite the presence of protease inhibitors in the lysis buffer, reproducible patterns of proteolysis were observed in the N-PbCS fusion protein, as previously observed [6]. [file 12936_2017_1908_MOESM1_ESM.pdf]

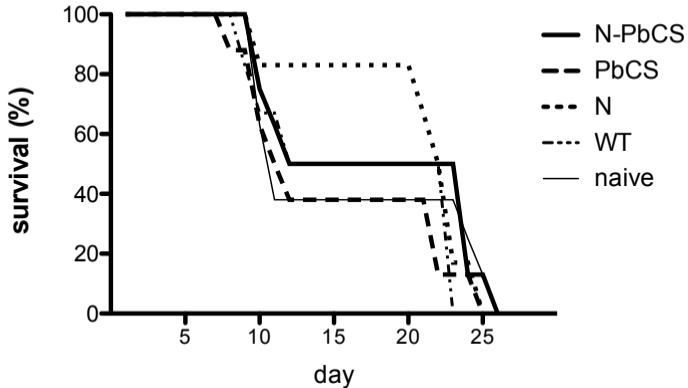

Supplement: Supplementary file 2 — Additional file 2: Figure S2. Survival curves of mice immunized with 30 YU of N-PbCS, PbCS, N or WT yeast lysates in Experiment I after challenge with GFP + Pb sporozoites. [file 12936_2017_1908_MOESM2_ESM.pdf]

# C

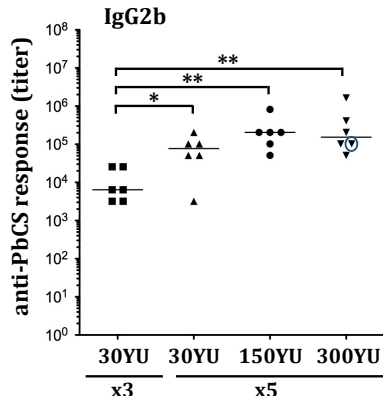

Supplement: Supplementary file 3 — Additional file 3: Figure S3. Isotyping of humoral IgG responses at day 42: IgG1 (A), IgG2a (B), and IgG2b (C) in mice immunized with 30, 150 and 300 YU of N-PbCS non-adjuvanted yeast lysates. Bars correspond to median values per group. “x3”-three bi-weekly immunizations; “x5”-five weekly immunizations. Mouse sera were analysed at 1/103 dilution. Asterisks (*) indicate significant median differences (one symbol for p < 0.05, two for p < 0.005, Mann–Whitney nonparametric test). Antibody titers of the non-parasitized mouse are encircled. [file 12936_2017_1908_MOESM3_ESM.pdf]

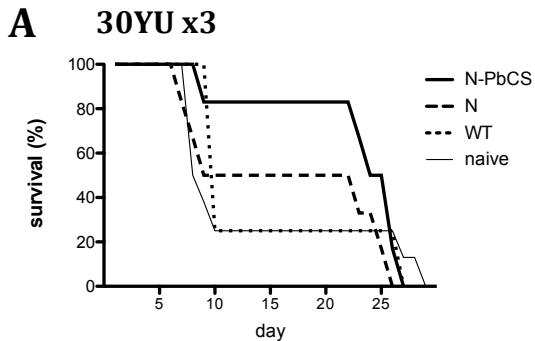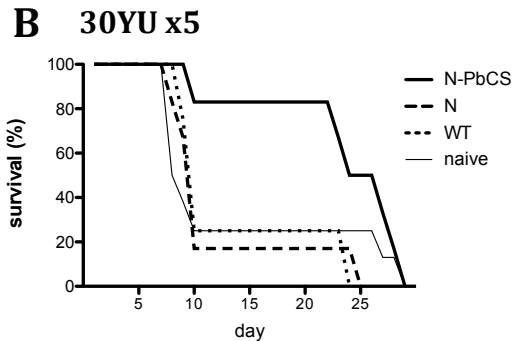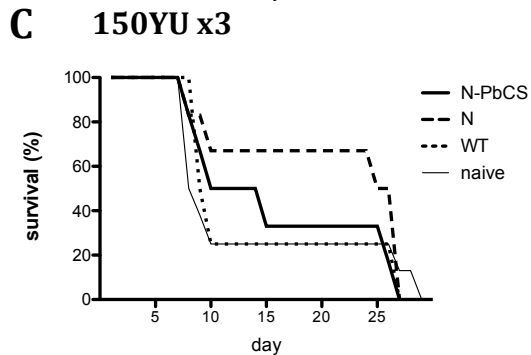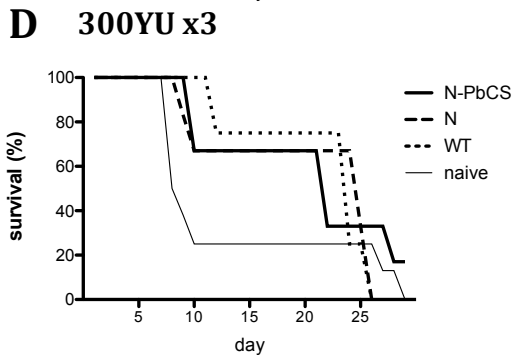

Supplement: Supplementary file 4 — Additional file 4: Figure S4. Survival curves of immunized mice in Experiment II after challenge with GFP + Pb sporozoites. (A)-30 YU N-PbCS, N or WT yeast lysates 3 times bi-weekly; (B)-30 YU N-PbCS, N or WT yeast lysates 5 times weekly; (C)-150 YU N-PbCS, N or WT yeast lysates 5 times weekly; (D)-300 YU N-PbCS, N or WT yeast lysates 5 times weekly. [file 12936_2017_1908_MOESM4_ESM.pdf]

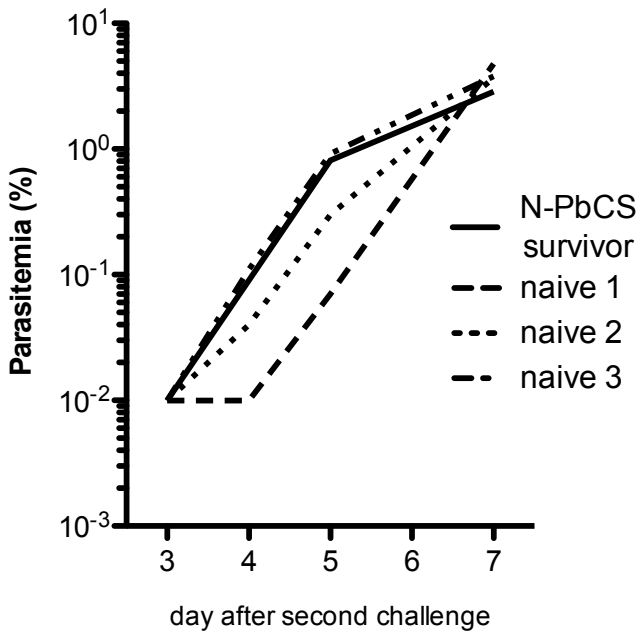

Supplement: Supplementary file 5 — Additional file 5: Figure S5. Second challenge of the mouse that survived the first challenge in Experiment II. Log10 values of parasitemia in the mouse immunized with 300 YU N-PbCS yeast lysate that survived first infection at day 42 (“N-PbCS survivor”) and three naive mice at day 5 following infection at day 118 with 104 GFP+ P. berghei sporozoites. [file 12936_2017_1908_MOESM5_ESM.pdf]

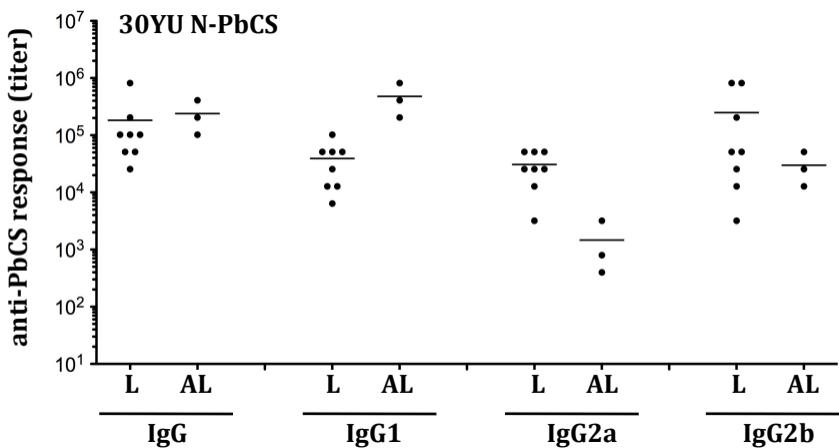

Supplement: Supplementary file 6 — Additional file 6: Figure S6. Isotyping of humoral IgG responses at day 42 in mice immunized with 30 YU N-PbCS yeast lysates. L-non-adjuvanted lysate formulation administered 5 times weekly, AL-alum-adjuvanted lysate formulation administered 3 times bi-weekly. Bars correspond to median values per group. [file 12936_2017_1908_MOESM6_ESM.pdf]

## N-PbCS lysates with alum

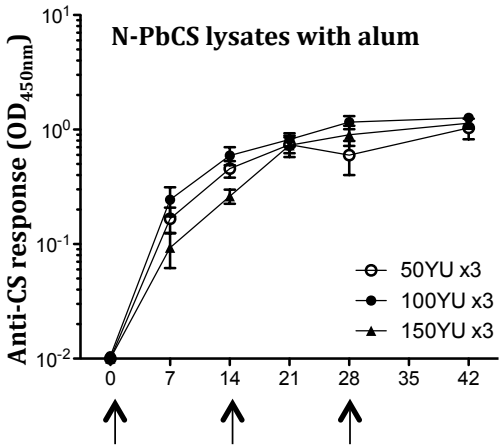

Supplement: Supplementary file 7 — Additional file 7: Figure S7. Kinetics of humoral anti-PbCS responses in immunized mice with N-PbCS yeast lysates adjuvanted with alum. OD450nm are expressed in log10 scale. Arrows indicate immunization schedule. Mouse sera were analysed at 1/103 dilution. [file 12936_2017_1908_MOESM7_ESM.pdf]

## A 50YU

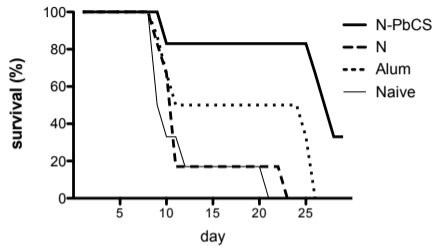

## B 100YU

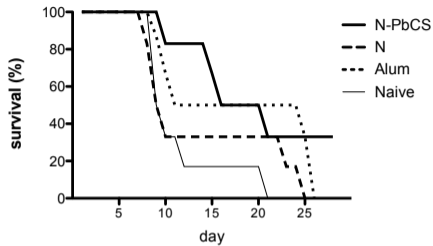

## C 150YU

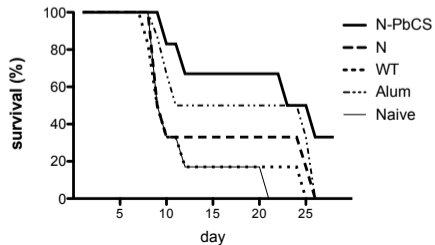

Supplement: Supplementary file 8 — Additional file 8: Figure S8. Survival curves of immunized mice in Experiment III after challenge with GFP + Pb sporozoites. Mice were immunized 3 times bi-weekly with (A)-50 YU N-PbCS or N yeast lysates; (B)-100 YU N-PbCS or N yeast lysates; (C)-150 YU N-PbCS, N or WT yeast lysates: in comparison to mice immunized with the adjuvant alone (Alum group) and naive mice. [file 12936_2017_1908_MOESM8_ESM.pdf]
